# The Nasser–Gavvala–Shirodkar–Botchu Classification: A Classification System for Loosening of Endoprosthetic Replacements

**DOI:** 10.3390/jcm14176300

**Published:** 2025-09-06

**Authors:** Ahmed Abdul Hadi Harb Nasser, Sai Niharika Gavvala, Kapil Shirodkar, Rajesh Botchu

**Affiliations:** 1 The Royal Orthopaedic Hospital, Birmingham B31 2AP, UK; ahmed.nasser@nhs.net; 2 Department of Musculoskeletal Radiology, The Royal Orthopaedic Hospital, Birmingham B31 2AP, UK; 3 Department of Radiology, Royal Lancaster Infirmary, Lancaster LA1 4RP, UK

**Keywords:** endoprostheses, arthroplasty, prosthetic implant

## Abstract

**Objectives:** The indications for femoral endoprosthesis replacement (EPR) use in limb reconstruction have broadened over the last decade. Despite its success, loosening remains the most common reason for failure. No previous system has classified loosening based on the anatomical site in relation to the prosthesis. The aim of this study is to propose a simple reproducible classification system for EPR loosening. **Methods:** Adult patients that underwent a revision EPR for loosening from 1 January 2023–1 May 2025 were included. Radiographs and computed tomography (CT) images were retrospectively reviewed. The grading was developed on radiographs to classify loosening around EPRs as normal (grade 1), loosening at the shoulder (grade 2), loosening around the shaft of the peg (grade 3), loosening below the tip of the prosthesis (grade 4), associated penetration of prosthesis through the cortex (grade 5), and associated fracture (grade 6). **Results:** A total of 28 patients were included. The majority of patients were male (n = 17; 61%) with a mean age of 50.6 years (SD 16.1). The average time from the index surgery to diagnosis of loosening was 10.1 years (SD 7.6). The most common pattern of loosening was grade 3 (N = 16; 57.1%). **Conclusions:** Our classification system proposes an easily adopted way to describe all patterns of loosening around EPRs, potentially guiding revision surgical strategies. Standardizing the approach in evaluating loosening will aid in producing national guidelines for managing this complex complication and may help improve future EPR design.

## 1. Introduction

Femoral endoprosthetic replacement (EPR) is a well-established surgical technique for malignant bone tumors [1,2,3,4]. The use of EPRs has become the method of choice for reconstruction after primary bone tumor resection around the femur [1]. Due to modern chemotherapy regimens, survival from primary bone tumors has increased, resulting in improved limb salvage surgical techniques. Since the 1980s, authors have demonstrated that EPRs used for bone tumors resulted in excellent functional outcomes with limb salvage rates exceeding 95% [1]. Plotz et al. [4] demonstrated that the probability of survival of the lower limb in patients that underwent reconstructive surgery with an EPR in the 20th century was 95% after 10 years. The 5-year survivorship of EPRs used for tumors in the distal femur has also increased from 20% to more than 85% in the last few decades [3].

More recently, the indications for EPR use have broadened to include metastatic bone disease, failed internal fixation, periprosthetic fractures, prosthetic joint infection (PJI), and revision total hip replacement (THR) or total knee replacement (TKR) with significant bone loss [5,6,7,8,9,10]. Traditional methods of stabilization, such as intramedullary nail fixation, were the most common techniques when surgically managing metastatic bone disease while tumor resection and use of EPR were reserved for a specific subset of patients [5]. However, recent EPR use in metastatic bone disease has been associated with favorable survival, earlier mobilization and weight bearing, decreased rates of return to theater, and decreased rates of implant failure when compared to fixation [5]. The primary advantage of EPR use in metastatic bone disease is that it allows a single definitive surgery which is of vital importance in such patients with immunocompromised status [5].

Apart from tumors, failed revision arthroplasty has recently become another challenging indication for the use of EPRs [6]. Previous studies have demonstrated that the burden of revision arthroplasty is on the rise and will continue to do so [7]. The biggest challenge in revision arthroplasty is managing significant bone loss to allow for a stable prosthesis [7]. Bone loss resulting from previous procedures and implant failure has previously been managed by impaction allograft, allograft-prosthesis composites, or excision arthroplasty [7]. Due to the limitations associated with allografts in tumor surgery, EPRs emerged as a viable management modality for patients with significant bone loss such as Paprosky defects of 3B or higher [7]. Sonia et al. [9] has demonstrated that proximal femoral replacements (PFRs) are salvage options for severe bone loss in complex revision total hip arthroplasty.

An increase in the use of EPRs has also been recently seen in the setting of geriatric distal femoral fractures. Previous studies have demonstrated the use of distal femoral replacements (DFRs) in comminuted intra-articular fractures, periprosthetic fractures with loose femoral components, fractures with inadequate bone stock for fixation, patients with fractures and pre-existing arthritis, and in cases of nonunion or malunion [10,11]. Proximal femoral EPR has also been reported to be beneficial in complex osteoporotic extracapsular hip fractures when a single procedure and early mobilization are required [7]. Although associated with some risks and complications when compared to fixation, EPRs are viable reconstructive options associated with high limb salvage rates. Treatment with ORIF for geriatric fractures often requires a prolonged period of restricted weight bearing which is non-beneficial in an already susceptible population [10]. A recent meta-analysis, comparing distal femoral replacements (DFRs) with open reduction and internal fixation (ORIF) in the management of distal femur periprosthetic fractures, demonstrated similar complication and revision rates [11]. In addition, DFR use in periprosthetic fractures has been associated with decreased operating times, decreased blood loss, and decreased length of stay when compared to fixation or revision arthroplasty with standard prosthesis [10]. Compared to arthrodesis and amputation, previously the only viable options in cases not amenable to fixation or standard revision arthroplasty, femoral EPRs are associated with improved functional and psychological outcomes, earlier weight bearing, and are more cost-effective [12,13].

The cost effectiveness of limb salvage with EPRs has previously been demonstrated for patients with bone tumors [12]. Although the cost of an EPR prosthesis is higher than performing an amputation, the actual costs and maintenance of artificial limbs increase exponentially over the years. Grimer et al. [12] demonstrated that the cost of an amputation for primary bone tumor is £5757 + 4705y ($US 9442 + 7716y), where y equals the number of years since the amputation, while the total cost of an EPR is £12 806 + £865 ($US 21,002 + 1419) per year. The cost saving of an EPR in the year 1997 for one patient over a 20-year period exceeds £70,000 [12]. This is mainly due to the considerable costs associated with a sophisticated artificial limb which will require multiple adjustments, maintenance, and the need for further replacements during a patients lifespan [12].

Despite the benefits, EPRs are associated with some risks and high complication rates [10]. These include prosthetic joint infection, aseptic loosening, wound complications, surgical site infections, periprosthetic fractures, extensor mechanism disruption, hematoma, nerve palsy, dislocation, and arthrofibrosis [10]. In a recent systematic review, revision rates of EPRs used for metastatic bone lesions in the proximal femur exceeded 10%, with fractures and aseptic loosening identified as major causes of revision [14]. In EPRs used for non-oncological conditions, local complication rates can exceed 20% while return to baseline mobility can be as low as 50% and one-year mortality rates can reach 9% [15]. The survivorship of EPRs used for non-oncological reasons at 2 to 5 years ranges between 60% and 80%, with aseptic loosening identified as a major cause of failure [9,10]. In a recent multi-center study investigating the clinical outcomes of distal femoral EPRs, aseptic loosening was amongst the highest three causes of local complications post operatively [15]. However, recent advances in the design of EPRs, such as the use of collars for improved osseointegration and silver coatings to decrease infection, have improved survivorship [15].

Various classification systems have attempted to classify EPR failure. The widely accepted Henderson classification identifies 5 types of failure, with aseptic loosening (type 2) identified as being the most common mode of failure in the literature [16]. Previous studies have demonstrated aseptic loosening rates of up to 17% in EPRs [17,18,19,20]. Hou et al. [21] further classified aseptic loosening of distal femoral EPRs based on the degree of loosening. However, this aseptic loosening classification system was purely focused on distal femoral replacements and did not include other femoral EPRs such as proximal femoral replacements. The causes and possible prevention strategies of aseptic loosening of prosthesis has recently been the focus of orthopedic clinical research [21]. The etiology of aseptic loosening is likely multifactorial. Factors that may predispose to aseptic loosening of EPRs include resection length, type of implant, size and diameter of implant and bone to stem ratio [22,23,24,25]. Longer stem and body lengths require longer resections and result in an increase in mechanical forces going through the tip of the stem, subsequently increasing the risk of aseptic loosening [15]. In addition, greater physical activity in younger patients with EPRs in situ combined with poor shock attenuation by the prosthesis can result in aseptic loosening [3]. These factors ultimately lead to subsequent failure and need for revision surgery, which presents a significant challenge to orthopedic oncologists and revision arthroplasty surgeons.

No system has yet classified loosening based on the anatomical site in relation to the prosthesis and biomechanics of loosening, which explains why the ideal revision strategy for loose EPRs remains challenging. The degree and site of loosening may have a direct impact on the subsequent amount of bone loss and can affect the residual length of intact bone. The anatomical site of loosening may also help explain the reasons behind EPR failure and may guide surgeons on the optimal revision strategy. Recognizing the site of loosening may also predict the subsequent mode of failure, subsequent bone loss, and consequences such as the pattern of periprosthetic fracture if left untreated. Due to the small number of cases performed and variation in EPR site and location, there is insufficient evidence on the optimal revision strategy for all EPRs.

In this study, we aimed to describe and illustrate a new easily reproducible classification system for the patterns of loosening around femoral EPRs. Our secondary objective was to describe the most common pattern of loosening around EPRs. This novel classification system may aid in improving future EPR design to avoid specific failure modes and to help guide management strategies.

## 2. Methods

A retrospective analysis was performed on a search of our Picture Archiving and Communication System (PACS) of adult patients that underwent EPR revision due to loosening from 1 January 2023 to 1 May 2025 by a single surgeon in a tertiary hospital. Patients that underwent an EPR revision due to tumor recurrence were excluded from the analysis. Radiographs and computed tomography (CT) images performed on a 64-slice Somatom AS (Siemens, Munich, Germany) were reviewed. An axial, sagittal, and coronal CT protocol with slice thickness of 0.6 mm was performed on patients lying supine. Long leg radiographs or femoral radiographs were obtained of the entire EPR, with at least 2 views (AP and lateral). Loosening was diagnosed clinically with pain on weight bearing and radiologically if widening of greater than 2 mm at the prosthesis-bone interface or the bone-cement interface was noted on imaging. Cases were analyzed by a fellowship consultant musculoskeletal radiologist, experienced fellowship-trained radiology specialist and an orthopedic surgeon. This study was registered as a local service evaluation (QI/2025-26/11) and ethical approval was not required.

**Figure 1:**
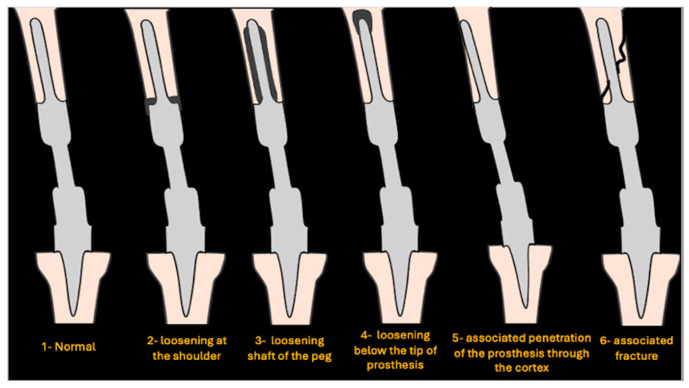
Schematic showing various grades of loosening.

After consensus between senior authors, a new simplified classification system was established. A total of 6 grades were developed on radiographs to classify loosening around EPRs as follows (Figure 1): normal (grade 1), loosening at the shoulder (grade 2), loosening around the shaft of the peg (grade 3), loosening below the tip of the prosthesis (grade 4), associated penetration of prosthesis through the cortex (grade 5), and associated fracture (grade 6). The imaging for each eligible case was reviewed by all authors and assigned a grade from the newly designed classification system.

**Figure 2:**
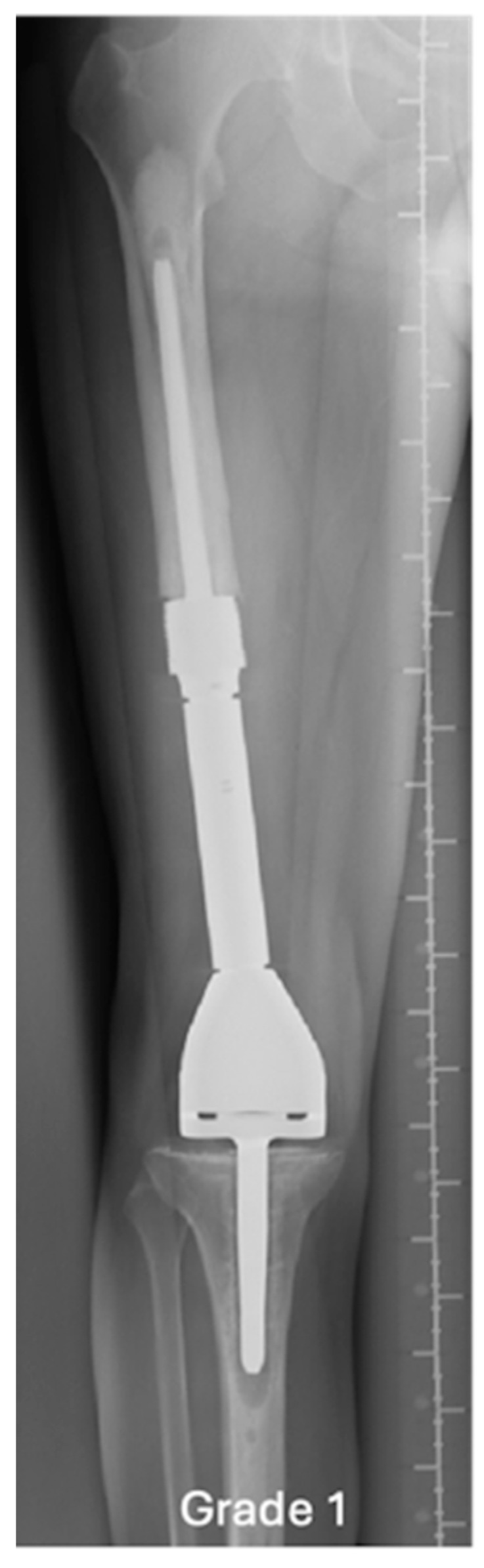
AP radiograph showing normal EPR, grade 1.

**Figure 3:**
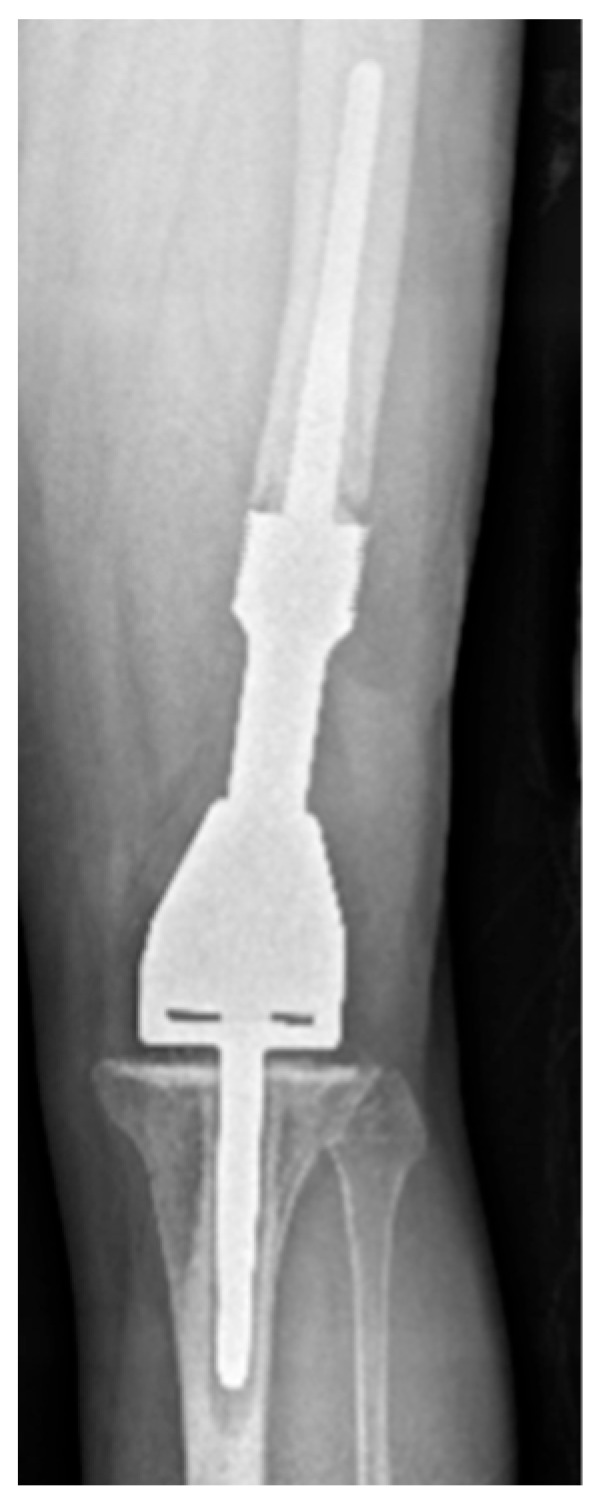
AP radiograph showing grade 2 loosening of EPR, loosening at the shoulder of femoral component.

**Figure 4:**
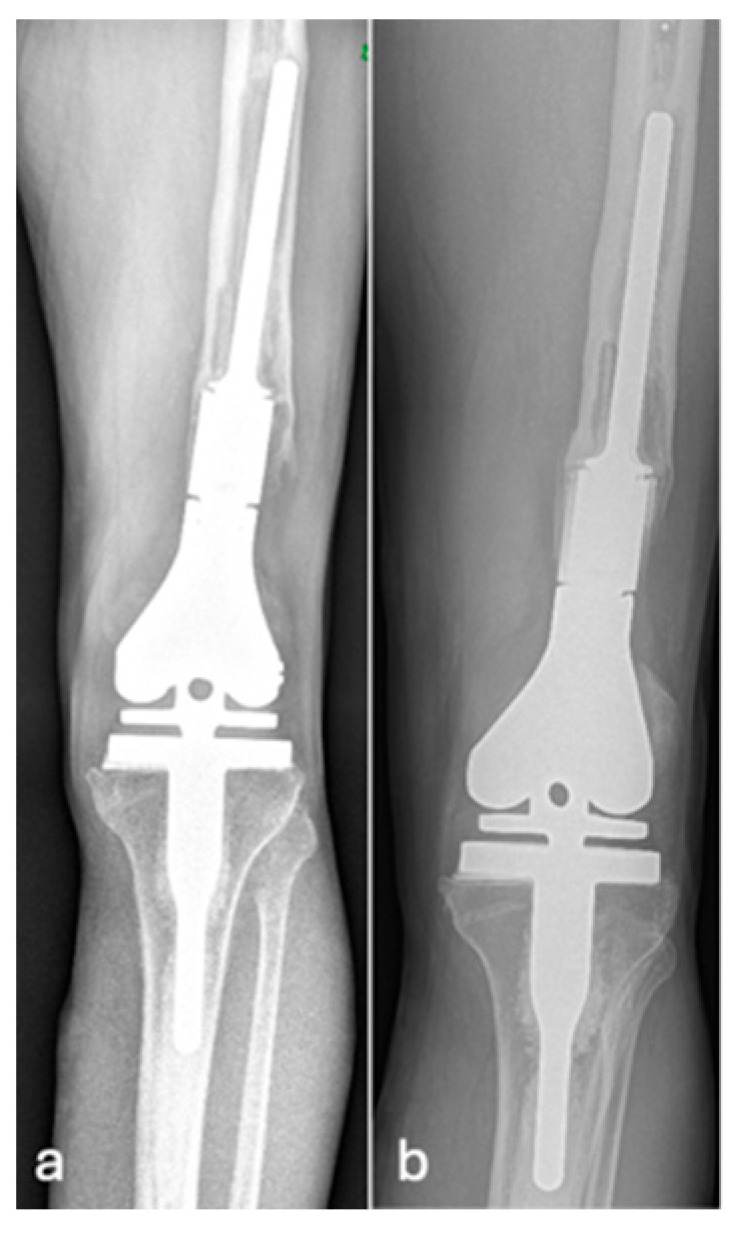
AP radiographs (**a**,**b**) showing grade 3 loosening of EPR, loosening around the peg of femoral component.

**Figure 5:**
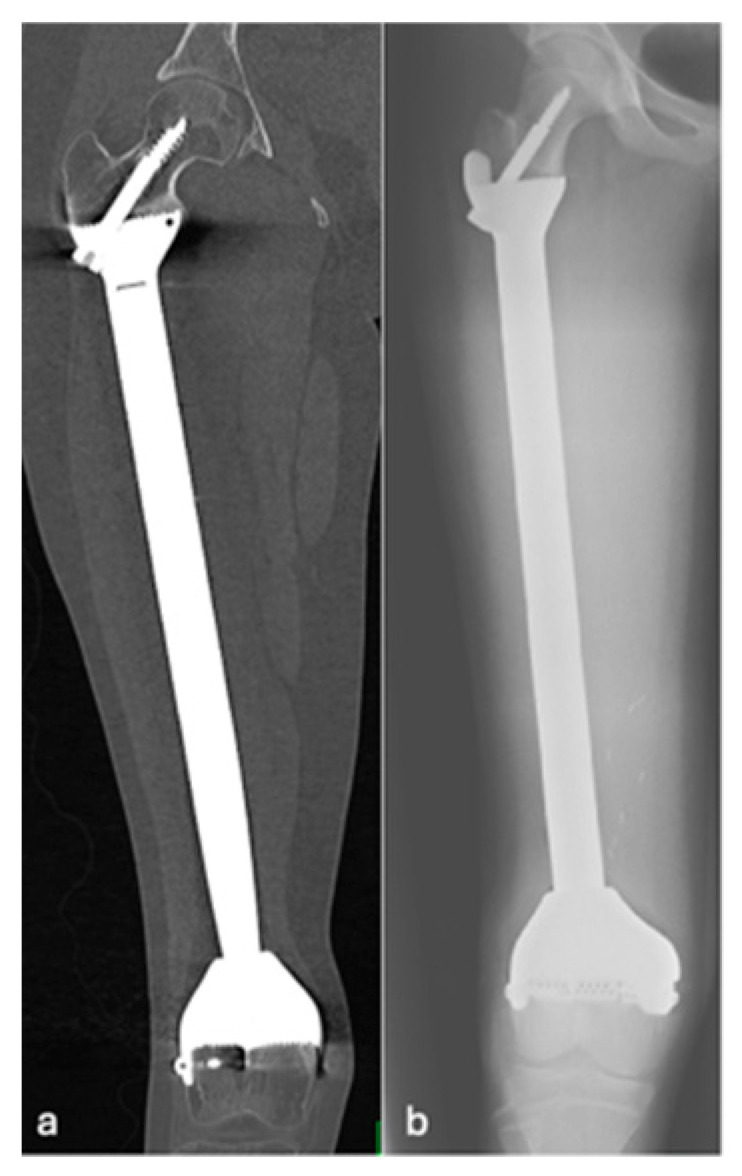
Coronal CT (**a**) and AP radiographs (**b**) showing grade 4 loosening of EPR with lucency around the proximal screw.

**Figure 6:**
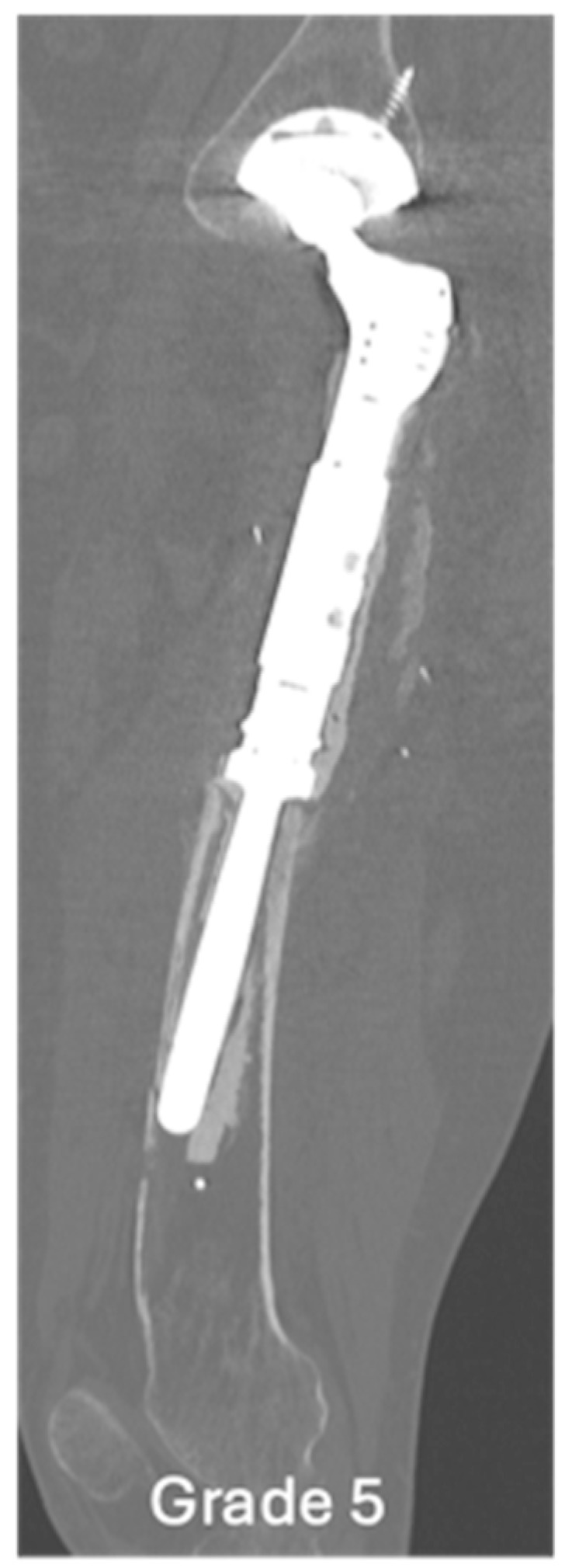
Sagittal CT showing grade 5 loosening of EPR, marked loosening of the femoral EPR with disruption of the anterior cortex.

**Figure 7:**
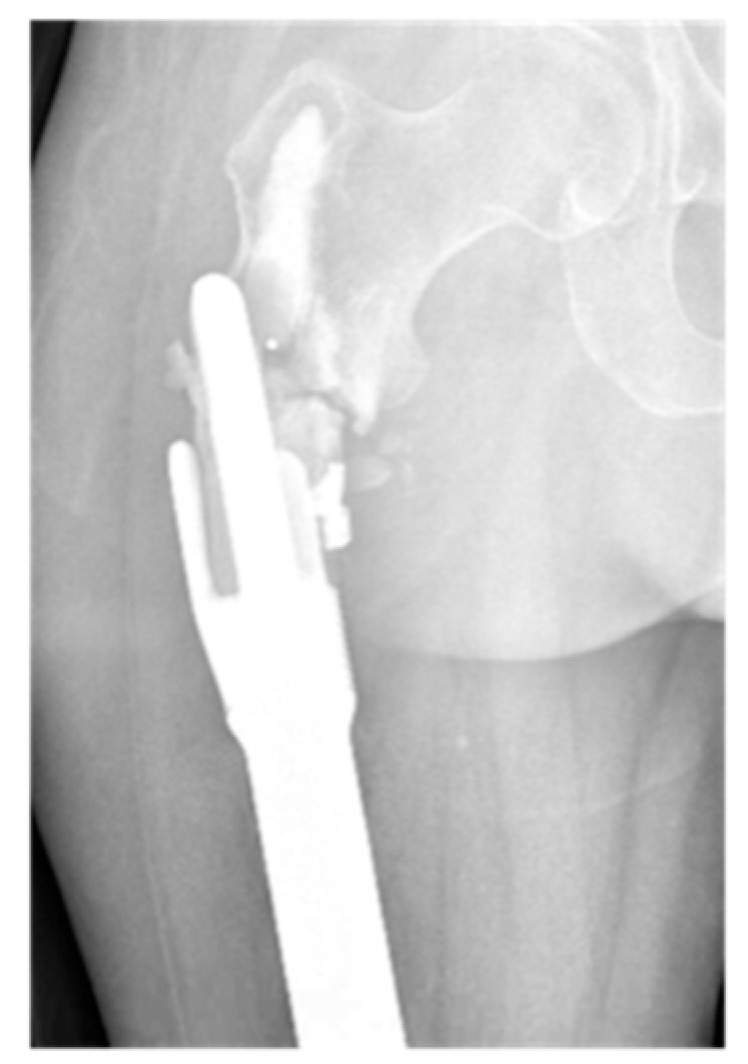
AP radiograph showing grade 6 loosening of EPR with periprosthetic fracture.

An example of a normal (grade 1) distal femoral EPR is demonstrated in Figure 2. Loosening at the shoulder of the prosthesis (grade 2) is illustrated in Figure 3. This pattern of loosening does not extend past the shoulder, with no evidence of loosening around the stem. Figure 4 demonstrates an example of loosening around the shaft of the peg (grade 3) while loosening below the tip of the prosthesis (grade 4) is demonstrated in Figure 5. Grade 4 pattern of loosening does not extend around the rest of the shaft of the peg. Associated penetration of the prosthesis through the cortex (grade 5) is illustrated in Figure 6. Associated fracture (grade 6) is demonstrated in Figure 7. Grade 6 describes any associated fracture pattern around the prosthesis.

Anonymized data was collected and stored in Microsoft Excel. Descriptive statistics was performed. Categorical variables were summarized as proportions. Continuous variables were presented as means. The Shapiro–Wilk test was used to determine the distribution of data in each variable.

## 3. Results

A total of 28 patients with evidence of femoral EPR loosening met the inclusion criteria. Table 1 describes the study population characteristics. In summary, the majority of patients were male (N = 17; 61%) with a mean age of 50.6 years (Range 23–82, SD 16.1). The average time from prosthesis insertion to the diagnosis of loosening was 10.1 years (Range 1–27, SD 7.6). The most common pattern of loosening was a grade 3 (N = 16; 57.1%). A total of 5 patients (17.9%) were classified as grade 2, 2 patients (7.1%) as grade 4, 1 (3.6%) as grade 5, and 4 (14.3%) as grade 6. All included patients had a pattern of loosening that fit a specific grade from the newly designed classification system.

The majority of EPRs that were in situ and loose were cemented implants (N = 25; 89%). Only 3 patients (11%) had loosening around uncemented prostheses. A total of 16 patients (57%) had an initial EPR for an oncological reason. All patients that underwent a revision after a primary EPR for an oncological reason were tumor free at the site of the prosthesis.

## 4. Discussion

The indications for EPR use have expanded considerably in the last few decades. Originally designed for malignant bone tumors, their use has expanded in management of metastatic bone disease and insufficient bone stock in fractures that fail reconstruction, PJI, and revision THR or TKR [5,6,7,8,9,10]. Grammatopoulos et al. [7] demonstrated salvage in all patients and recommends the continued use of EPRs for non-neoplastic indications including PJI. Femoral EPRs improve overall functional and psychological outcomes when compared to arthrodesis and amputation [12]. Despite its success, loosening around EPRs is common and must be identified early on to avoid the associated morbidity. Progression of loosening results in further bone loss. Therefore, early detection may allow for the initiation of less invasive treatment modalities avoiding the use of a total femoral replacement.

To the best of our knowledge, this is the first classification system that incorporates all the patterns and consequences of loosening around EPRs. Previous classification systems have attempted to classify modes of failures of EPRs. Henderson et al. [16] classified EPR failure as soft tissue failure (type 1), aseptic loosening (type 2), structural failure (type 3), infection (type 4), and tumor progression (type 5). Although this widely used classification is well adopted, aseptic loosening which is the most common mode of failure of EPRs was only subclassified according to the time frame when the complication occurred. The site around which aseptic loosening has occurred, which may explain the consequence of the loosening, was not previously described in depth. Henderson et al. [16] further demonstrated that aseptic loosening was the most common mode of failure in the literature and that the relative incidences of the five primary modes of EPR failure were dependent on anatomic location. However, their classification system categorized failure according to the mode and not the anatomical site. Hou et al. [21] further classified the degree of loosening around distal femoral EPRs as a prosthesis with no displacement (type 1), displaced prosthesis (type 2), length of the medullary cavity below the highest horizontal line of the isthmus can be used for fixation < 5 cm/2 times the diameter of the medullary cavity (type 3), and lost isthmus medullary cavity or defects involving the metaphysis (type 4). Hou et al. [21] further expanded on management strategy by advocating for revision surgery for type 1 and 2, the use of custom short stem prosthesis for type 3, and a custom short stem prosthesis combined with a lateral steel plate and screws to preserve the original hip joint structure. However, this classification system focused on the degree of loosening in distal femoral EPRs only and did not take categorize failures according to the anatomical site. No previous system has incorporated associated fractures or classified loosening according to the anatomical site in relation to any EPR implant, which is vital in guiding surgeons on the optimal management strategy.

Various factors that affect loosening around femoral EPRs have been previously described. The type of prosthesis, cement usage during implantation, constraint, the diameter of the stem used, shape of the stem, coating around the stem, length of bony resection, and bone to stem ratio are factors that may cause specific patterns of loosening around femoral EPRs [22,23,24,25,26,27,28]. Ogura et al. [22] has previously investigated the independent predictors of overall failure and cause-specific failure in patients undergoing DFRs. Percentage of femoral resection and extent of quadriceps muscle resection were independent predictors of overall DFR failure, while patient age and percentage of femoral resection were significantly associated with mechanical failure [22]. The type of joint resection was the only independent factor associated with DFR infection [22]. The extent of quadriceps muscle resection, fixation of femoral component, and a bone stem ratio of 2.5 were all demonstrated to be significant independent predictors of aseptic loosening of DFRs [22]. Kinkel et al. [29] has also previously reported that uncemented distal femoral replacement using the Modular Universal Tumour and Revision System (MUTARS) (Implantcast, Buxtehude, Germany) have a significantly lower aseptic loosening rate when compared to cemented implants at 46 months (9% versus 25%, respectively).

Various treatment strategies have been proposed to manage EPR loosening. Prosthetic joint infection (PJI) must first be ruled out since radical debridement, which is essential for success, can change the definitive management strategy drastically [30]. The European Bone and Joint Infection Society (EBJIS) has clear diagnostic criteria for PJI [31]. However, PJI can present similar to aseptic loosening both clinically and radiologically. The use of serum biomarkers may have an important role when differentiating PJI from aseptic loosening [32]. Various surgical strategies have also been described for managing loosening without PJI or post eradication of infection. Gaitan-Lee et al. [33] advocates for revision EPR augmentation with polymethylmethacrylate and a condylar plate. Other techniques described include the use of bone allografting, wire mesh and impaction bone grafting, and total femur EPR [34,35,36]. A high degree of variation exists in the literature regarding the optimal management approach to EPR loosening.

Total femoral arthroplasty (TFA) is a reconstructive procedure that has been previously described for a variety of indications when significant femoral bone loss is present. Aseptic loosening could subsequently result in massive femoral bone loss if left untreated, necessitating the use of a TFA. The use of TFAs as a salvage option will persist due to the projected increase in the number of revision arthroplasty procedures worldwide [37]. Although it is considered a salvage option avoiding the need for an amputation, the complication rates are significant. Owen et al. [38] has recently demonstrated that the ten-year survival free rate of any revision after a TFA performed for non-oncological reasons is 34%. The reasons for revision included prosthetic dislocation, PJI, and aseptic loosening [38]. A recent systematic review including more than 1000 patients has also demonstrated a failure rate of 34% in TFAs used for oncological and non-oncological reasons [39]. The potential for aseptic loosening of the tibial component of the TFA and mechanical failure tends to also increase over time [39]. Therefore, although limb salvage can be achieved with its use, TFA should be reserved as a last resort for complex cases that fail reconstruction with more bone preserving alternatives. We believe that loosening around EPRs should be diagnosed early to prevent further bone loss that may necessitate the use of a TFA. Patients should be followed up closely and an early bone conserving management strategy should be sought. Classifying loosening around EPRs based on anatomy is vital when managing these complex cases.

We believe our classification system comprehensively describes all the patterns of loosening around EPRs and may guide surgeons when deciding on the best definitive management strategy. In our classification system, grade 2 is described as loosening at the shoulder, with intact bone distally around the stem. Although revision surgery may be required in certain cases, sufficient bone stock more distally will usually allow stable fixation with adjuncts such as bone grafting or revision stems. A grade 3 describes loosening around the shaft of the peg. Although the degree of loosening around the shaft of the peg may vary, this pattern may result in the prosthesis becoming inherently unstable and eventually piercing through the cortex. Early detection is vital in preventing further bone loss in the future and avoiding major reconstructive surgery with total femoral endoprosthesis. Grade 4 describes loosening below the tip of the prosthesis. This may cause metaphyseal bone loss resulting in a challenging situation requiring custom implants and total femur replacement. Grade 5 describes associated penetration through the cortex, which may be managed with a longer stem if sufficient bone exists. Finally, grade 6 describes an associated fracture around the EPR, which may be managed with fixation or revision depending on the stability of the original prosthesis.

We acknowledge that our recommendations on management strategy for each grade in our classification system may not apply to all cases of aseptic loosening. The management of loosening around EPRs with residual or recurrence of tumor differs drastically from tumor free cases. Cases that need further tumor resection may require further bony resections, resulting in insufficient bone stock for bone preserving revision surgery. However, all revised patients in our series were non tumor cases or tumor free at the time of revision surgery. Our classification system which describes the aseptic loosening pattern and site can however apply to any EPR performed for any indication because it focuses on the pattern and site of loosening and not necessarily the indication of surgery. Our classification system can apply to proximal femoral replacements or distal femoral replacements. It may also be possible to use this system for loosening around tibial or humeral EPRs, but this has not yet been validated or investigated in long bones apart from the femur.

Our study has several limitations due to its retrospective nature. No statistical analysis was performed due to the small sample size of patients. In addition, the proposed classification system is based on imaging. This study was also performed at a single center, with no long-term outcome measures reported after revision surgery. Finally, the degree and pattern of loosening identified in our patient cohort may have changed over time before the detection of loosening on the images.

## 5. Conclusions

Our proposed classification system can be easily adopted to describe most patterns of loosening around EPRs. Future studies should aim to validate this classification system in the future. This may help in standardizing the approach in the evaluation of loosening, the most common mode of failure in EPRs, which may aid in producing national and international management guidelines to decrease the variation in management of EPR loosening. This will also aid in improving future EPR design.

## Figures and Tables

**Table 1:** Table showing study population characteristics, the various grades of EPR loosening and time to loosening in our cohort.

	N (%)
Population size	28
Age, years	
Mean (SD)	50.57 (16.14)
Sex	
Male	17 (61)
Female	11 (39)
Time to loosening, years	
Mean (SD)	10.1 (7.55)
Grade of loosening	
Grade 2	5 (17.9)
Grade 3	16 (57.1)
Grade 4	2 (7.1)
Grade 5	1 (3.6)
Grade 6	4 (14.3)

## Data Availability

The data that supports the findings of this study are available to other researchers from the corresponding author upon reasonable request.
